# Evidence for measurement bias of the short form health survey based on sex and metropolitan influence zone in a secondary care population

**DOI:** 10.1186/s12955-020-01318-y

**Published:** 2020-04-03

**Authors:** Jake Ursenbach, Megan E. O’Connell, Andrew Kirk, Debra Morgan

**Affiliations:** 1 Department of Psychology, University of Saskatchewan, 9 Campus Drive, Saskatoon, S7N 5A5 Saskatchewan Canada; 2 College of Medicine, University of Saskatchewan, 9 Campus Drive, Saskatoon, S7N 5A5 Saskatchewan Canada; 3 Canadian Centre for Health and Safety in Agriculture, University of Saskatchewan, 9 Campus Drive, Saskatoon, S7N 5A5 Saskatchewan Canada

**Keywords:** Equivalency, Test bias, Psychometric theory, Quality of life

## Abstract

**Background and objectives:**

The 12-item Short Form Health Survey (SF-12) is a widely used measure of health related quality of life, but has been criticized for lacking an empirically supported model and producing biased estimates of mental and physical health status for some groups. We explored a model of measurement with the SF-12 and explored evidence for measurement invariance of the SF-12.

**Research design and methods:**

The SF-12 was completed by 429 caregivers who accompanied patients with cognitive concerns to a memory clinic designed to service rural/remote-dwelling individuals. A multi-group confirmatory factor analysis was used to compare the theoretical measurement model to two empirically identified factor models reported previously in general population studies.

**Results:**

A model that allowed mental and physical health to correlate, and some items to cross-load provided the best fit to the data. Using that model, measurement invariance was then assessed across sex and metropolitan influence zone (MIZ; a standardized measure of degree of rurality).

**Discussion:**

Partial scalar invariance was demonstrated in both analyses. Differences by sex in latent item intercepts were found for items assessing feelings of energy and depression. Differences by MIZ in latent item intercepts were found for an item concerning how current health limits activities.

**Implications:**

The fitting model was one where the mental and physical health subscales were correlated, which is not provided in the scoring program offered by the publishers. Participants’ sex and MIZ should be accounted for when comparing their factor scores on the SF-12. Additionally, consideration of geographic residence and associated cultural influences is recommended in future development and use of psychological measures with such populations.

## Background and objectives

As the proportion of older adults in Canada grows, it is crucial that that healthcare services in Canada adapt to meet their needs [1]. One important way healthcare and other policy decisions are made involves assessing individuals’ health-related quality of life, which is a construct that summarizes their physical, social, and emotional status as it relates to their prior and current health state [2]. One commonly used measure of health related quality of life is the Medical Outcomes Study 12-item Short Form Health Survey (SF-12) [3].

The items of the SF-12 were derived from the 36-Item Short Form Health Survey (SF-36), a longer health survey which has been used in more than 5000 studies internationally [4] that has consistently demonstrated utility in distinguishing between known groups based on physical or mental health status [3]. The SF-36 has eight subscales covering a range of physical and mental health concerns, such as ‘role limitations due to physical health’ and ‘emotional well-being.’ The subscale scores are combined to produce a physical health summary component (PCS) and a mental health summary component (MCS), which are based on a principal component analysis of the eight subscale scores using an orthogonal rotation, based on an assumption that physical and mental health are not correlated [3]. The stated goal of the SF-12 was to reproduce the PCS and MCS scores in a survey that could be completed in under two minutes. To this end, the SF-12 items were selected using stepwise regressions of the MCS and PCS on the SF-36 items in a large population study conducted in the United States, which produced regression weights for 12 of the SF-36 items that best approximated the subscale scores of the test. This model approximation approach to test length reduction is effective in some situations, but may not generalize well to different populations [5].

Despite its widespread use, two prominent concerns about the validity of the SF-12 have been raised. First, some researchers argue that the measurement model of the scale, although theoretically described by Ware et al. [3], was not actually tested in the creation of the scale and is not empirically supported [6]. This criticism relates to the way that the summary scores for the mental and physical health subscales were derived, specifically, to the extent that the measurement model was derived from the SF-36, and the assumption that the latent constructs of physical and mental health are uncorrelated [7, 8]. Instruments such as the SF-36 and the SF-12 consist of a series of Likert-scale questions which act as indicators, or manifest variables, influenced by unobserved, or latent variables, which for the SF-12 are the mental and physical health related quality of life. Several studies have employed an exploratory factor analysis approach using a principal component analysis with orthogonal rotation, consistent with the development of the SF-36 and the assumption of uncorrelated mental and physical health. While some of these studies supported the hypothesized 2 component structure [9–11], others produced a three-component solution, with a general health component in addition to the mental and physical health components [12, 13]. While the evidence of a two-factor solution is supportive of the theoretical model, significant methodological issues limit the strength of this evidence, specifically, the estimated correlations between the individual items and the latent physical and mental health variables are likely inaccurate due to the use of principal component analysis, which assumes an initial communality of one, and the specified orthogonal relationship between the mental and physical health components, which is not supported empirically. The assumption of an orthogonal relationship has resulted in biased scoring coefficients where poorer physical health results in an overestimate of mental health and vice versa [6, 14–16].

Other studies have used alternative approaches to determine the appropriate measurement model of the SF-12. The models identified by two such studies are summarized in Table 1. In the first study, Fleishman and Lawrence [17] used a confirmatory factor analysis (CFA) to explore the factor structure by beginning with the simple structure outlined by Ware et al. [3], and then incrementally improving it by allowing the factors to correlate and using the modification indices. They achieved an adequate fit by allowing the residuals on several similarly worded items to correlate, as well as allowing certain items to cross-load on both factors. In the second study, Tucker et al. [7] followed a similar approach but did not permit items to cross load. In summary, although the SF-12 generally appears to have a two-factor solution, the measurement model specified by Ware and colleagues does not seem well supported empirically. Various alternative models have been identified in general population studies, and it is not clear which is appropriate for the subpopulation of the present study.

**Table 1:** Sample Characteristics

*Characteristic*	
Sample size (n)	429
Age, *M* (*SD*)	70.9 (0.49)
Sex, *Female* (%)	238 (58.8%)
MIZ, *no MIZ to weak MIZ* vs *moderate to urban* (%)	191 (47.2%)
SF-12, *M* (SD)	44.49 (7.43)
PCS, *M* (SD)	20.23 (4.67)
MCS, *M* (SD)	24.20 (3.86)

The second concern is that the physical and mental health related quality of life estimates of the scale are not invariant, or in other words, are biased against some populations, and therefore group comparisons are not appropriate unless measurement invariance is established first. That is, does the SF-12 measures these latent variables in the same manner for all persons who complete the scale, or does it measure these latent variables differently for subpopulations [18]. When an instrument demonstrates measurement invariance, knowledge of population membership will provide no new information about an individual’s scores on the observed variables given knowledge of their level of the latent variable [18]. Measurement invariance is difficult to assess because it is impossible to directly know an individual’s level of the latent variable. For example, men and women could demonstrate different mean scores on the MCS scale, which could represent true differences in mental health status, differences in measurement model but equality in mental health status, or a combination of the two.

One approach to assessing measurement invariance involves assessing items individually for differential item functioning. Some studies have found evidence of significant differential item functioning across groups on the SF-12. For example, in a nationally representative sample study conducted in the United States, Flieshman and Lawrence [17] found evidence that men were less likely to endorse items suggesting they had trouble climbing stairs, they felt downhearted, or they lacked energy when compared to women of a similar mental and physical health status. Similar problems with differential item functioning have been reported when comparing White to Black and Hispanic American respondents, and comparing younger to older respondents [17, 19]. In contrast, some studies have investigated differential item functioning across age and sex and have not found evidence of it [20], while others have found evidence of it when comparing patients with stroke to normal controls, but argued that the evidence present failed to reach the level of practical significance [21].

A second approach to assessing measurement invariance involves using multigroup confirmatory factor analysis (MG-CFA) to compare the change in model fit indices across a series of models which progressively constrain the structural model to be equal across groups [18]. As the models become incrementally more restrictive, a hierarchy of invariance has been established [22]. The most commonly assessed forms of invariance are: *Configural*, where groups have the same number of latent variables, and items load on latent variables in a similar pattern; *Metric* (weak factorial), where item loadings do not significantly differ across groups; *Scalar* (strong factorial), where item intercepts do not significantly differ across groups; and *Strict factorial*, where item residual variances do not differ across groups [22]. In order to meaningfully compare group means on the latent variable, scalar invariance is recommended [18].

A few studies of the SF-12 have been conducted using this methodology, though predominantly in clinical or marginalized groups. For example, Okonkwo et al. [21] found evidence of metric invariance between patients who experienced stroke and healthy controls. Similarly, another study found evidence of strict invariance between four groups of Canadians with different levels of vulnerable housing status [23]. However, the measurement invariance of the SF-12 has never been investigated in rural- versus urban-dwelling populations.

Many rural populations have higher rates of mortality, disability, and chronic disease than urban-dwelling populations [24]. Some factors contributing to this disparity are structural, such as a low population density leading to transportation issues and a lack of access to specialists [24, 25]. Others have suggested that cultural differences, such as an increased emphasis on self-reliance, may contribute to health disparities as well, with the caveat that rural populations are a heterogenous group that varies along a continuum of acculturation [26, 27]. Indeed, the idea that rural populations may be culturally distinct from urban populations has a long history and empirical support [28–30]. It is possible that cultural differences in some rural populations may result in biased estimates of health status when using the SF-12, for example, some rural-dwelling individuals may place an emphasis on self-reliance, which could contribute to systematic underreporting of symptoms.

Establishing measurement invariance is a prerequisite for test interpretation and group comparison [18]. As the SF-12 is used not only for research, but to inform public policy [8], failure to attend to these measurement issues has potentially costly or harmful consequences. We will investigate the measurement invariance of the SF-12’s MCS and PCS subscales across sex and geographical proximity to metropolitan areas. As evidence of differential item functioning by sex has been demonstrated in previous studies [17], we hypothesize that the SF-12 will not demonstrate metric invariance. However, in the previous study, although the magnitude of loadings differed, the number of factors and pattern of loadings was consistent between men and women. As such we hypothesize that the SF-12 will demonstrate configural invariance. Finally, although there is evidence calling into question the validity of the SF-12 with minority populations, its psychometric properties in a rural-dwelling general population have not been previously investigated, so there is no direct evidence to support a directional hypothesis. For that reason, a directional hypothesis is not made regarding the measurement invariance for a rural versus urban-dwelling dementia/cognitive concern caregiver population.

## Research design and methods

### Participants

The analyses in this study were conducted using archival data collected at the Rural and Remote Memory Clinic (RRMC) in Saskatoon, Saskatchewan. The clinic services a predominantly rural patient population (with rurality defined as living at least 100 km outside of the two major urban centers in Saskatchewan). Patients are referred for further investigation of memory or other cognitive or behavioral concerns. Participants in this study were a cohort of individuals who accompanied patients to the clinic, typically family members, hereafter referred to as caregivers. Exclusion criteria included inability to read and write in English and mental or physical disability that precluded completion of the questionnaires. They completed a questionnaire packet which included the SF-12 while the patient they accompanied was assessed. Further information about data collection and other RRMC operations are detailed elsewhere [31]. Research ethics approval was provided by the University of Saskatchewan Research Ethics Board (REB BEH 03–1219).

### Measures

The SF-12 has demonstrated evidence for reliability and validity in numerous populations and settings [3, 9–12, 21, 32], however, some concerns have been raised about the nature of the scoring algorithm and its validity with minority groups [7, 8, 17, 19]. As part of the initial validation of the instrument, Ware et al. [3] re-analyzed many cross sectional and longitudinal studies including the SF-36 using only the SF-12 items, and successfully reproduced the same pattern of results using the shorter survey. Some studies which included only the SF-12 items have been conducted in clinical and general populations and have demonstrated various forms of validity evidence such as known group [9, 11], convergent [9, 12, 21] and discriminant validity [32]. Test-retest reliability estimates have been reported at one week (PCS = .79, MCS = .79) [11] and two weeks (PCS = .86–.89, MCS = .76–.77) [3]. Point estimates for internal consistency have been reported that range from .80 to .87 for the PCS and .74 to .82 for the MCS [10, 21, 32]. Validity evidence has been presented for the SF-12 with a variety of populations, including a general population in the US [3, 17], Australia [7], Canada, Bermuda, New Zealand, and various European nations [8], among individuals with severe mental illness [11], patients in primary care [9], with stroke [21], Parkinson’s Disease [13], with post-partum women [19], among older Canadian Mennonites [10], and homeless/vulnerably housed Canadians [23].

Participants’ degree of rurality was quantified by using the Metropolitan Influence Zone (MIZ) that corresponded to their area of residence as reported by Statistics Canada. Geographical areas in Canada outside metropolitan areas are divided into different levels of MIZ according to the proportion of the employed workforce that commute into metropolitan areas as opposed to working locally [33]. For the present analyses, participants were divided into two groups: Low- to weak-MIZ (less than 5% of employed workforce commute) compared with moderate-MIZ to urban (greater than or equal to 5% commute or live in urban centers). The cut point was chosen to facilitate comparison with other studies using this population [34].

### Statistical procedure

The analyses were conducted using R version 3.4.2 [35]. Missing data were assessed using Little’s MCAR test to determine whether the missing data were missing completely at random (i.e., missing independently of other variables, both observed and unobserved) [36]. In the event that Little’s MCAR test was significant, dummy variables were created coding missingness for each observed variable to determine if data were missing at random (MAR) or missing not at random (MNAR). These dummy variables were then tested for independence from the remaining observed variables (sex and MIZ) using separate chi square tests and evaluated for significance at a Bonferroni-adjusted *p*-critical value of .002, where a significant result indicates that data for that item are conditional on other observed variables (MAR), whereas the absence of significant results for that item suggest that the missing data are conditional on an unobserved variable (MNAR). Although no attempt to impute missing values was planned, the nature of the missing data has important implications for the results that are taken up in the discussion.

Data were visually inspected for univariate normality using quantile-quantile plots. Then skewness and kurtosis statistics were calculated, divided by the standard error of the estimate, and evaluated against a critical *z* value of 1.96. Multivariate normality was assessed using Mardia’s Test of Multivariate Skewness and Kurtosis. In the event of nonnormally distributed data, mean- and variance-corrected weighted least squares (WLSMV) estimation was planned to account for the violated assumption where appropriate in the remaining analyses.

Descriptive statistics were reported using independent samples *t*-tests, Pearson’s correlations, and Fisher’s Z-Tests where appropriate for between-group comparisons. The measurement model of the SF-12 was then determined in this population by using CFA to compare the fit of the hypothesized model to empirically supported SF-12 models in other populations. Specifically, the model described by Ware et al. [3] was compared to others [7, 17]. Model fit was assessed based on the model fit criteria recommended by Hu and Bentler [37], with a comparative fit index (CFI) > .95, and a root mean squared error of approximation (RMSEA) < .06. The authors note that multiple fit indices should be considered when determining if fit is adequate and note that the RMSEA tends to be overly conservative in smaller sample sizes. The robust fit indices described above have demonstrated adequate capacity to detect model misspecifications in simulation studies of nonnormally distributed data when evaluated using Hu and Bentler’s criteria [38]. If none of the pre-specified models fit well, the modification indices were consulted and the parameter most contributing to poor fit was iteratively freed and model fit re-assessed until adequate. Based on the measurement model, Cronbach’s alpha coefficients and confidence intervals were estimated for the PCS and MCS within groups by sex and MIZ.

Once the measurement model was established, two analyses of measurement invariance were conducted based on sex and MIZ. In both cases, a grouping variable was coded with the demographic difference. First, configural invariance was assessed by fitting a multigroup CFA based on the measurement model previously determined in which item means, loadings, intercepts, and residuals were estimated freely. Adequate model fit according to the Hu and Bentler [37] guidelines provides evidence that the scale supports configural invariance. Subsequent forms of invariance were evaluated by comparing the change in CFI from the less constrained to more constrained model, where a significant deterioration in model fit is indicated by change in RMSEA > .01 and/or change in CFI < −.004 [39, 40]. Metric invariance was first assessed by constraining item slopes to be equal between groups and comparing the change in CFI from the configural to metric model. If metric invariance was supported, scalar invariance was then assessed by also constraining item intercepts to be equal and comparing the change in CFI from the metric to scalar model. Similarly, if scalar invariance was supported, strict invariance was also assessed by constraining item residual variances to be equal and comparing change in CFI from scalar to strict model. If invariance was not supported at any level, constraints were iteratively released based on the modification indices to determine partial invariance [18].

## Results

Of the 544 participants in the initial sample, 21.1% were missing data regarding either their MIZ or one or more items on the SF-12, resulting in a final sample size of 429. The most common pattern of missingness was participants who omitted all items on the SF-12, accounting for 16.7% of the missing data. Little’s MCAR Test was significant, χ^2^(244) = 333.18, *p* < .001, indicating data were not MCAR, suggesting that missing data were conditional on another variable. None of the follow-up chi square tests were significant at the Bonferroni adjusted p-critical value when testing the independence of each item’s missingness from Sex and MIZ, suggesting that the missing data were conditional on an unobserved variable, or MNAR.

Univariate normality was assessed visually and statistically. All SF-12 items showed significant univariate skew (*p* < .05), and all but items 1, 2, 5, 8, 11 were significantly kurtotic (*p* < .05). Mardia’s test of multivariate skewness and kurtosis was significant for both skewness (*b* = 30.03, *z* = 2147.26, *p* < .001) and kurtosis (b = 297.50, z = 19.49, *p* < .001). As the data were not normally distributed WLSMV estimation was used.

Descriptive statistics of the sample are reported in Table 1. The average age of participants was 70.9 years (*SD* = 0.5). Most participants were female, and about half of the sample resided in a low to weak MIZ area. Across the full sample, participants’ mean raw score on the SF-12 was 44.5 (*SD* = 7.4). Women (*M* = 45.4, *SD* = 7.0) scored significantly higher than men (*M* = 43.2, *SD* = 7.8), *t* (364.2) = − 3.04, *p* = .003. Participants scores from a no- to low-MIZ area (*M* = 44.3, *SD* = 7.7) did not significantly differ from those from a moderate-MIZ to metropolitan area (*M* = 44.6, *SD* = 7.2), *t* (416.9) = 0.38, *p* = .708. Within groups estimates of internal consistency were calculated with 95% confidence intervals for the SF-12 overall, and then for MCS and PCS separately. In both between group comparisons, the Cronbach’s alpha confidence intervals overlapped, suggesting that internal consistency did not significantly differ by sex or MIZ. In addition, all estimates exceeded 0.70, providing evidence of adequate internal consistency. The estimates were all significant moderate negative correlations, which ranged from −.56 for women to −.57 for men, and from −.57 for no- to low-MIZ to −.61 for moderate MIZ to urban-dwellers. Fisher’s Z test was not significant for sex, *Z* = 0.14, *p* > .05, or MIZ, *Z* = 0.63, *p* > .05, indicating that the correlations did not significantly differ in either comparison.

To establish a baseline measurement model, several increasingly complex models were compared, as shown in Table 2. Model 1, described by Ware et al. [3] but with correlated factors, did not provide an adequate fit to the data. Similarly, Model 2, reported by Flieshman and colleagues [17] also did not fit the data well. Model 3, described by Tucker and colleagues [8] also failed to produce an adequate fit. A model specification search required two iterative consultations of the modification indices and freeing of parameters to produce an adequately fitting model. The final model used for the measurement invariance analysis consisted of correlated physical and mental health factors, and items 1,2,3,4,5,8,10,12 on the physical health subscale and items 1,4,5,6,7,9,10,11,12 on the mental health subscale, with residual covariances for item pairs 5–6, 7–8, 9–10, 12–13, 12–14.

**Table 2:** Comparison of model fit for baseline model

	χ2 (df)	*p* value	RMSEA [95% CI]	CFI	TLI
Model 1: Ware et al. with correlated factors	311.71 (53)	<.001	.107 [.095–.118]^a^	.790^a^	.739
Model 2: model 1 + cross-loading items 1, 10, 12	198.31 (50)	<.001	.083 [.071–.096]^a^	.880^a^	.841
Model 3: model 2 + residual covariances for items from same SF-36 scale	161.93 (46)	<.001	.077 [.064–.090]^a^	.906^a^	.865
Model 4: model 3 + cross-loading items 4, 5 and residual covariance for items 9, 10	83.24 (43)	<.001	.047 [.031–.062]	.967	.950

Model 1: items 1,2,3,4,5,8 on physical health factor and items 6,7,9,10,11,12 on mental health factor

All models estimated using WLSMV

^a^Poor model fit indicated by RMSEA > .05 and CFI < .95

The SF-12 demonstrated partial scalar invariance with regard to sex as indicated in Table 3. All relevant model parameters were invariant to sex with two exceptions. The latent intercepts for items 10 and 11 varied by sex, shown in Table 4. The sample means for item 11 (feeling depressed) were 4.18 for females and 3.90 for males, for an observed mean difference of 0.28, with higher numbers suggestive of greater depressive symptomatology. The latent intercept estimates for that item were 4.011 for males and 4.178 for females, a difference of 0.167. These results indicate that of the 0.28 observed mean difference, 0.167 is due to the difference in intercept, suggesting that the minor difference in male and female responses on the item is partly due to influences other than the mental and physical health factors modelled here. In contrast, while observed group means for item 10 (feeling energetic) were similar, with 3.46 for males and 3.45 for females, the estimated latent intercepts differed, with 3.628 for males and 3.453 for females, a difference of 0.175, suggesting that despite similar observed means, female scores were associated with slightly lower factor scores relative to male scores due to influences outside the factors modeled here.

**Table 3:** Measurement invariance tests regarding sex and Metropolitan Influence Zone

	Model	χ2	df	RMSEA [95% CI]	CFI
Sex	1. Configural	120.002	86	.043 [.022–.060]	.971
	2. Metric	118.349	101	.028 [.000–.047]	.985
	3. Scalar	139.229	111	.035 [.010–.051]	.976*
	3a. Partial Scalar (intercepts for items 10,11 free)	129.661	109	.030 [.000–.048]	.982
	4. Partial Strict (intercepts for items 10,11 free)	141.434	121	.028 [.000–.046]	.983
MIZ	1. Configural	118.580	86	.042 [.021–.060]	.973
	2. Metric	122.457	101	.032 [.000–.050]	.982
	3. Scalar	138.409	111	.034 [.008–.051]	.977*
	3a. Partial Scalar (Intercepts for item 2 free)	135.244	110	.033 [.000–.050]	.979
	4. Partial Strict (Intercepts for item 2 free)	163.871	122	.040 [.022–.055]*	.965*

*significant deterioration in model fit indicated by change in RMSEA > .01 and/or change in CFI < −.004 [40]

**Table 4:** Factor model parameter estimates from partial strict model across sex^a^

	Factor Loadings			
Item	Physical	Mental	Unique Variances	Latent Intercepts
1. General health	1.000^b^	0.204	0.453	3.430
2. Current health limits moderate activities	0.804		0.250	2.555
3. Climbing stairs	0.830		0.293	2.450
4. Accomplishing less	1.157	0.484	0.548	4.017
5. Health limits kinds of activities	1.446	0.298	0.427	4.167
6. Emotional problems accomplishing less		1.000^b^	0.397	4.305
7. Emotional problems being less careful		0.902	0.242	4.520
8. Pain interferes with work	1.342		0.478	4.218
9. Feeling calm		0.505	0.491	3.669
10. Feeling energetic	0.588	0.361	0.499	Male: 3.628 Female: 3.453
11. Feeling depressed		0.574	0.666	Male = 4.011 Female = 4.178
12. Social activities	0.528	0.579	0.449	4.468

^a^All parameters reported in unstandardized form

^b^Parameter fixed to 1 for identification

The SF-12 demonstrated partial scalar invariance with respect to MIZ in this sample. Factor loadings and latent intercepts were equal between groups except for the intercept for item 2 (Current health limits moderate activities), as indicated in Table 5. On that item, participants from the Moderate-MIZ/Urban group had an observed mean of 2.47, while those from the No−/Weak-MIZ group had a mean of 2.53, for an observed mean difference of 0.06. Separate latent intercepts were estimated for that item for each group, with the Moderate-MIZ/Urban group estimated at 2.426 and the No−/Weak-MIZ group at 2.532, for a difference of 0.106, suggesting that while the observed mean item scores for the two groups are very similar, No−/Weak-MIZ group scores were associated with slightly higher physical health scores for reasons not captured by the factor model.

**Table 5:** Factor model parameter estimates from partial strict model across MIZ^a^

	Factor Loadings		
Item	Physical	Mental	Latent Intercepts
1. General health	1.000^b^	0.204	3.316
2. Current health limits moderate activities	0.774		No/Weak MIZ: 2.532 Moderate MIZ/Urban: 2.426
3. Climbing stairs	0.812		2.363
4. Accomplishing less	1.220	0.484	3.869
5. Health limits kinds of activities	1.521	0.298	4.001
6. Emotional problems accomplishing less		1.000^b^	4.231
7. Emotional problems being less careful		0.900	4.449
8. Pain interferes with work	1.284		4.083
9. Feeling calm		0.510	3.626
10. Feeling energetic	0.565	0.342	3.441
11. Feeling depressed		0.610	4.060
12. Social activities	0.546	0.549	4.361

^a^All parameters reported in unstandardized form

^b^Parameter fixed to 1 for identification

## Discussion

Our first hypothesis, that the SF-12 would demonstrate only configural invariance, was not supported. Rather, our results indicate that the SF-12 demonstrates partial scalar invariance across sex, suggesting that it may not be appropriate to compare the MCS and PCS scores of males and females in a rural, secondary care dementia/cognitive concern caregiver population. There is a difference in latent intercept estimates by sex of 0.17 on item 11 (feeling depressed, 5-point Likert) favoring females, and a difference of 0.18 on item 10 (feeling energetic, 5-point Likert) favoring males. These differences are of similar magnitude but in opposite directions, and therefore may balance out. Although previous studies have not examined the measurement invariance of the SF-12 across sex using this methodology, most studies of differential item functioning have found no evidence of practically significant differences in the way scale items function for men compared with women. For example, one study conducted with a Parkinson’s disease patient population used an item response theory analysis and reported that items appeared to function similarly for men and women and concluded that comparisons across sex were appropriate [20]. Another study found evidence that some items functioned differently for men and women in a stroke population, but concluded that the significant results were attributable to the large sample size and did not reach the level of practical significance [21]. As previously discussed, one study did find evidence of significant, meaningful differential item functioning by sex in a nationally representative sample. The authors attributed this to a male tendency to avoid responding in a way that indicates weakness or dependence. They noted that this interpretation was supported in their sample by men’s statistical reticence to endorse items suggesting difficulty climbing stairs, lacking energy, or feeling downhearted [17].

Regarding our second hypothesis, we did not specify a priori whether we anticipated measurement invariance on the SF-12 across MIZ. The SF-12 demonstrated partial scalar invariance with respect to MIZ in this sample. The intercept for item 2 (Current health limits moderate activities) differed across groups by 0.11 favoring the No−/Weak-MIZ group, suggesting their responses were associated with slightly higher physical health factor scores for reasons not captured by the factor model. One other study has investigated the psychometric properties of the SF-12 in a rural setting, specifically among older adult rural-dwelling Mennonites in Canada [10]. Although they did not examine invariance directly, they found evidence of validity in that population. Specifically, using an exploratory factor analysis, they found the expected two-factor solution, and they found evidence of known group validity on a range of groups such as age, income, marital status, self-reported health, social interaction, and spirituality. These results are generally consistent with the present study as they suggest that the SF-12 may be validly used in some rural-dwelling populations. Consistent with our findings of limited invariance across MIZ, a previous study using a subpopulation of the same dementia/cognitive concern caregiver population found evidence of only configural and weak invariance across MIZ on the Zarit Burden Inventory [34], a measure of dementia caregiver burden. Taken together, these results suggest that factors related to MIZ influence the measurement properties of psychometric instruments. Consideration of participants’ geographic residence and associated cultural influences is recommended in future development and use of psychological measures with such populations.

There are some limitations to this study beyond the issue of estimation and the violation of the assumption of multivariate normality previously discussed. Specifically, approximately one in five participants were missing data and could not be included. Subsequent analysis suggested that the data were MNAR, or in other words, conditional on an unobserved variable. Although it is not clear from the data extracted from the archival dataset why these data were missing, it is possible that those participants who did not provide data did so because the SF-12 was not a valid instrument for them, which would limit the generalizability of these results. For example, it is possible that a culturally distinct subpopulation of dementia/cognitive concern caregivers felt disenfranchised due to a history of negative experiences in Canadian social programs and therefore chose not to participate in data collection. While multiple imputation is typically recommended when data are MAR and even MNAR [41], in this case there is a risk that doing so will obscure systematic differences in the missing subpopulation yet provide the illusion of methodological rigour. Caution is urged in the generalization of these findings because in addition to the high proportion of missing data, the target population is quite unique, specifically, it is comprised of largely rural-dwelling caregivers of people with cognitive concerns referred to secondary care.

In future research it is important to replicate these findings in other urban and rural populations, ideally using nationally representative samples to minimize sampling bias. Other studies of the SF-12 have provided evidence of differential item functioning in various populations. Future research should examine the functioning of individual items in this dementia/cognitive concern caregiver population across different demographic variables to ensure that group comparisons are not biased. Finally, this study provided further evidence that the physical and mental health subscales of the SF-12 are correlated, suggesting that use of scoring coefficients that assume an orthogonal relationship produces inaccurate estimates of mental and physical health related quality of life [6, 14–16].

In conclusion, the current study adds to existing literature about the SF-12 by demonstrating the inadequacy of the measurement model proposed by Ware et al. [3] in a rural-dwelling dementia/cognitive concern caregiver population. It also providing evidence for partial scalar measurement invariance of the SF-12 across sex and MIZ, indicating that within this population, some caution should be used when comparing the physical and mental health related quality of life between those groups using the SF-12.

### Implications

Foremost, these data suggest the commercially available scoring program that models the mental health and physical health quality of life as orthogonal is not the best fit to the data. Although these results should be replicated, our findings have implications for use of the commercially available scoring program for the SF-12. Participants’ sex and MIZ should be accounted for when comparing their factor scores on the SF-12. Additionally, consideration of geographic residence and associated cultural influences is recommended in future development and use of psychological measures with such populations.

## Data Availability

Upon request.

## Abbreviations

SF-12: Short Form Health Survey
MIZ: Metropolitan influence zone
SF-36: 36-Item Short Form Health Survey
PCS: Physical health summary component
MCS: Mental health summary component
CFA: Confirmatory factor analysis
MG-CFA: Multigroup confirmatory factor analysis
RRMC: Rural and Remote Memory Clinic
REB BEH: Research Ethics Board
MAR: Missing at random
MNAR: Missing not at random
WLSMV: Variance-corrected weighted least squares
CFI: Comparative fit index
RMSEA: Root mean squared error of approximation
